# *Nepenthes* Ethyl Acetate Extract Provides Oxidative Stress-Dependent Anti-Leukemia Effects

**DOI:** 10.3390/antiox10091410

**Published:** 2021-09-02

**Authors:** Wangta Liu, Li-Ching Lin, Pei-Ju Wang, Yan-Ning Chen, Sheng-Chieh Wang, Ya-Ting Chuang, I-Hsuan Tsai, Szu-Yin Yu, Fang-Rong Chang, Yuan-Bin Cheng, Li-Chen Huang, Ming-Yii Huang, Hsueh-Wei Chang

**Affiliations:** 1 Department of Biotechnology, Kaohsiung Medical University, Kaohsiung 80708, Taiwan; liuwangta@kmu.edu.tw; 2 Department of Radiation Oncology, Chi-Mei Foundation Medical Center, Tainan 71004, Taiwan; 8508a6@mail.chimei.org.tw; 3 School of Medicine, Taipei Medical University, Taipei 11031, Taiwan; 4 Chung Hwa University Medical Technology, Tainan 71703, Taiwan; 5 Department of Biomedical Science and Environmental Biology, PhD Program in Life Science, College of Life Science, Kaohsiung Medical University, Kaohsiung 80708, Taiwan; u106023025@gap.kmu.edu.tw (P.-J.W.); u107023010@gap.kmu.edu.tw (Y.-N.C.); u107851101@gap.kmu.edu.tw (S.-C.W.); u107023007@gap.kmu.edu.tw (Y.-T.C.); s0932961465@gmail.com (I.-H.T.); t16094064@ncku.edu.tw (L.-C.H.); 6 Graduate Institute of Natural Products, Kaohsiung Medical University, Kaohsiung 80708, Taiwan; u105531007@gap.kmu.edu.tw (S.-Y.Y.); aaronfrc@kmu.edu.tw (F.-R.C.); 7 Department of Marine Biotechnology and Resources, National Sun Yat-sen University, Kaohsiung 80424, Taiwan; jmb@mail.nsysu.edu.tw; 8 Department of Radiation Oncology, Kaohsiung Medical University Hospital, Kaohsiung 80708, Taiwan; 9 Department of Radiation Oncology, Faculty of Medicine, College of Medicine, Kaohsiung Medical University, Kaohsiung 80708, Taiwan; 10 Institute of Medical Science and Technology, National Sun Yat-sen University, Kaohsiung 80424, Taiwan; 11 Center for Cancer Research, Kaohsiung Medical University, Kaohsiung 80708, Taiwan

**Keywords:** *Nepenthes*, leukemia cells, antioxidant, DNA damage, apoptosis, oxidative stress

## Abstract

Several kinds of solvents have been applied to *Nepenthes* extractions exhibiting antioxidant and anticancer effects. However, they were rarely investigated for *Nepenthes* ethyl acetate extract (EANT), especially leukemia cells. The purpose of the present study was to evaluate the antioxidant properties and explore the antiproliferation impact and mechanism of EANT in leukemia cells. Five standard assays demonstrated that EANT exhibits antioxidant capability. In the cell line model, EANT dose-responsively inhibited cell viabilities of three leukemia cell lines (HL-60, K-562, and MOLT-4) based on 24 h MTS assays, which were reverted by pretreating oxidative stress and apoptosis inhibitors (*N*-acetylcysteine and Z-VAD-FMK). Due to similar sensitivities among the three cell lines, leukemia HL-60 cells were chosen for exploring antiproliferation mechanisms. EANT caused subG1 and G1 cumulations, triggered annexin V-detected apoptosis, activated apoptotic caspase 3/7 activity, and induced poly ADP-ribose polymerase expression. Moreover, reactive oxygen species, mitochondrial superoxide, and mitochondrial membrane depolarization were generated by EANT, which was reverted by *N*-acetylcysteine. The antioxidant response to oxidative stress showed that EANT upregulated mRNA expressions for nuclear factor erythroid 2-like 2 (*NFE2L2*), catalase (*CAT*), thioredoxin (*TXN*), heme oxygenase 1 (*HMOX1*), and NAD(P)H quinone dehydrogenase 1 (*NQO1*) genes. Moreover, these oxidative stresses led to DNA damage (γH2AX and 8-hydroxy-2-deoxyguanosine) and were alleviated by *N*-acetylcysteine. Taken together, EANT demonstrated oxidative stress-dependent anti-leukemia ability to HL-60 cells associated with apoptosis and DNA damage.

## 1. Introduction

Leukemia is a type of cancer that generates abnormal, immature blood cells derived from bone marrow dysfunction and carcinogenesis. Leukemia is classified into acute and chronic types with different cell differentiation [1]. The acute types contain acute myeloid leukemia (AML) [2] and acute lymphoblastic leukemia (ALL) [3]. AML is common in adults, and ALL is common in children [4]. Cancer Statistics from 2019 show that leukemia in males and females rank ninth and tenth, respectively, for estimated new cases, and high mortality ranks sixth and eighth, respectively, for estimated deaths [5]. Leukemia therapy includes the combined strategy of chemotherapy, radiation, targeting, and bone marrow transplantation [6]. However, there are side effects commonly associated with leukemia therapy [7]. To avoid or reduce those, it is necessary to develop novel drugs for leukemia treatment.

Many natural and cultivated hybrids of pitcher plant species belonging to *Nepenthes* (tropical carnivorous plants) are traditionally applied as herbal medicines in Southeast Asia [8]. *Nepenthes* extracts were reported to inhibit bacterial and fungal growth [9] and inflammation [10]. Different *Nepenthes* extracts showed antiproliferation effects in several cancer cells, but their effects were rarely reported for leukemia cells. For example, the methanol extract of *N. alata* Blanco can inhibit breast cancer cell proliferation [11]. Similarly, our previous findings showed that ethyl acetate extracts of *N. thorelii* × *(ventricosa* × *maxima)* (EANT) induced antiproliferation and reactive oxygen species (ROS) in breast cancer cells [12]. However, the potential antiproliferation effect of EANT on leukemia cells remained uninvestigated.

Many ROS-modulating agents were developed to induce oxidative stress in anticancer therapy by excessing the ROS tolerance in cancer cells [13,14,15,16,17]. The rationale is that drug-induced excessive oxidative stress frequently triggers DNA damages [18] and apoptosis [19]. In addition, antioxidants exhibiting dual functions for regulating cellular oxidative stress have been reported [20]. Therefore, a high concentration of antioxidants may induce oxidative stress. However, the antioxidant effect of EANT remains unclear.

The objectives of the present study are to assess the antioxidant ability and antiproliferation effects of EANT on leukemia cells. In addition, the detailed mechanism for anti-leukemia of EANT is also explored in terms of viability, apoptosis, oxidative stress, and DNA damage detection.

## 2. Materials and Methods

### 2.1. EANT Extraction and Chemicals

The extracting condition, detailed high-performance liquid chromatography (HPLC) fingerprint information, and bioactive compounds of EANT were reported previously [12]. Briefly, the air-dried aerial parts of *N. thorelii* × (*ventricosa* × *maxima*) were immersed in methanol and subsequently partitioned by water/ethyl acetate. Finally, the ethyl acetate layer was collected and called EANT further on.

Inhibitors for ROS and apoptosis, including *N*-acetylcysteine (NAC) [21] and Z-VAD-FMK (ZVAD) [22], were obtained from Sigma-Aldrich (St. Louis, MO, USA) and Selleckchem (Houston, TX, USA). EANT and ZVAD were melted in dimethyl sulfoxide (DMSO), where NAC was dissolved in double-distilled water.

### 2.2. Determination of 2,2-Diphenyl-1-Picrylhydrazyl (DPPH), 2,2-Azinobis (3-Ethyl-Benzothiazoline-6-Sulfonic Acid) (ABTS), Hydroxyl Radical Scavenging Activities

DPPH [23] and ABTS^•+^ [24,25] scavenging activities of EANT were detected as described. In brief, 125 μM DPPH in ethanol or ABTS reagents (7.4 mM ABTS^•+^ and 2.6 mM persulfate) were added (1:1) to EANT (0–1000 μg/mL in DMSO). After 30 and 10 min, the absorbances of DPPH and ABTS responses were recorded at 517 and 734 nm utilizing a multiplate reader (Bio Tek; Winooski, VT, USA). The results are reported as % of control cell signals.

Hydroxyl radical scavenging activity of EANT was detected as described with slight modification [26]. In brief, EANT (100 μL, 0–1000 μg/mL in DMSO) was added to 2.5 mM 2-deoxyribose/0.2 M phosphate-buffered saline buffer (690 μL, pH 7.4) and 1.04 mM EDTA/0.1 mM ferric chloride solution (100 μL). Ascorbic acid (100 μL, 10 mM) and H_2_O_2_ (10 μL, 0.1 M) were further added for 10 min incubation at 37 °C. Then, thiobarbituric acid (500 μL, 1%) was mixed with trichloroacetic acid (1 mL, 2.8%), reacted at 100 °C for 8 min, and ice chilled for 20 min. After standing at room temperature for 10 min, the absorbances of hydroxyl radical scavenging responses were recorded at 532 nm utilizing a multiplate reader. The results are reported as % of control cell signals.

### 2.3. Ferric Ion Reducing Antioxidant Power (FRAP) and Ferrous Ion Chelating Power (FCP) Assays

The FRAP activity of EANT was detected as described [24]. In brief, phosphate buffer (0.2 M, pH 6.6) and potassium ferricyanide (1%) were equally mixed (100 vs. 100 μL) and incubated with EANT (100 μL, 0–1000 μg/mL in DMSO) for 20 min at 50 °C. Then, TCA (100 μL, 10%), deionized water (400 μL), and ferric chloride (400 μL, 0.1%) were added for a 10 min treatment. The absorbance of FRAP response was recorded at 705 nm by means of a multiplate reader, and the results were reported as % of control cell signals.

The FCP activity of EANT was analyzed as described [24]. In brief, deionized water (740 μL) and ferrous chloride (20 μL, 2 mM) reacted with EANT (200 μL, 0–1000 μg/mL in DMSO). Then, ferrozine (40 μL, 5 mM) was added with modest shaking for 10 min. The absorbance of the FCP response was recorded at 562 nm utilizing a multiplate reader. The results are reported as % of control cell signals.

### 2.4. Cell Lines and Viability

Three ATCC (Manassas, VA, USA) human leukemia cell lines were chosen, namely AML (acute promyelocytic HL-60 [27] and chronic myelogenous K-562 [28]), and ALL (T-cell acute lymphocytic MOLT-4 [23]) types. They were cultured with RPMI medium (Gibco; Grand Island, NY, USA) and mixed with 10% FBS and antibiotics (50 U/mL penicillin and 50 μg/mL streptomycin). MTS-based CellTiter 96^®^ AQueous One Solution Cell Proliferation Assay (Promega Corporation, Madison, WI, USA) was applied to assess cell viability. In addition, the multiplate reader detected MTS response at 492 nm [29].

### 2.5. Cell Cycle Analysis

By staining with 7-aminoactinmycin D (7AAD; 1 μg/mL) for 30 min (Biotium; Hayward, CA, USA), cell cycle phases were routinely determined by cellular DNA content using a flow cytometer [30]. In addition, the Accuri C6 flow cytometer (Becton-Dickinson, Mansfield, MA, USA) was used, and the cell cycle phase was analyzed by BD Accuri C6 software Version 1.0.264.21.

### 2.6. Apoptosis Analysis

Apoptosis was detected through annexin V expression using the annexin V/7AAD kit [31] (Strong Biotech; Taipei, Taiwan) and caspase-Glo^®^ 3/7 assay (Promega; Madison, WI, USA) [32]. According to the user manual, annexin V intensities were measured by an Accuri C6 flow cytometer (Becton-Dickinson, Mansfield, MA, USA). In addition, the caspases 3/7 activity of EANT-treated cells was detected by a luminometer (Berthold Technologies GmbH & Co., Bad Wildbad, Germany) and adjusted by their cell viabilities.

Apoptosis was also detected by Western blotting as described [22]. Apoptosis signaling proteins were detected by using the primary antibodies against cleaved poly (ADP-ribose) polymerase (c-PARP) and c-Caspase 3 (c-Cas 3) (Cell Signaling Technology, Inc., Danvers, MA, USA). mAb-β-actin antibody was chosen as an internal control (Sigma-Aldrich; St. Louis, MO, USA).

### 2.7. Flow Cytometric Analysis to Detect ROS, Mitochondrial Superoxide (MitoSOX), and Mitochondrial Membrane Potential (MMP)

ROS, MitoSOX, and MMP content were measured by the radial-detecting probes of 2′,7′-dichlorodihydrofluorescein diacetate (DCFH-DA; Sigma-Aldrich, St. Louis, MO, USA) [33] (10 μM, 30 min), MitoSOX™ Red [34] (50 nM, 30 min), and DiOC_2_(3) [35] (Invitrogen; San Diego, CA, USA) (5 nM, 30 min). Their signals were detected using an Accuri C6 flow cytometer.

### 2.8. Real-Time RT-PCR Analysis to Detect Antioxidant-Related Gene Expressions

RNA extraction and reverse transcription-PCR were performed [36]. Using the touch-down PCR program [37], the mRNA expressions for antioxidant-related genes [38], such as nuclear factor erythroid 2-like 2 (*NFE2L2*), catalase (*CAT*), thioredoxin (*TXN*), heme oxygenase 1 (*HMOX1*), and NAD(P)H quinone dehydrogenase 1 (*NQO1*), were evaluated by reference to the *GAPDH* gene. The 2^−ΔΔCt^ method [39] was applied for gene expression calculation. All the detailed primer information was mentioned previously [38].

### 2.9. Flow Cytometric Analysis to Detect DNA Damage Markers (γH2AX and 8-Hydroxy-2-Deoxyguanosine (8-OHdG))

γH2AX [14] and 8-OHdG [40] were detected through their targeted antibodies, and their levels were assessed by the flow cytometer as described. The primary antibodies (4 °C, 1 h) of γH2AX and the 8-OHdG-FITC antibody (Santa Cruz Biotechnology; Santa Cruz, CA, USA) were applied to 75% ethanol-fixed cells. 7AAD (5 μg/mL, 30 min) was further added for γH2AX detection.

### 2.10. Statistical Analysis

Using JMP 12 software (SAS Campus Drive, Cary, NC, USA), the significance was assessed by one-way analysis of variance (ANOVA) with HSD post hoc test in multiple comparisons. Data showing different alphabets at the top reveal significant differences.

## 3. Results

### 3.1. Dose-Response Effect of EANT on Scavenging Activities of DPPH, ABTS, and Hydroxyl Radical, as well as FRAP and FCP Activities

**Figure 1:**
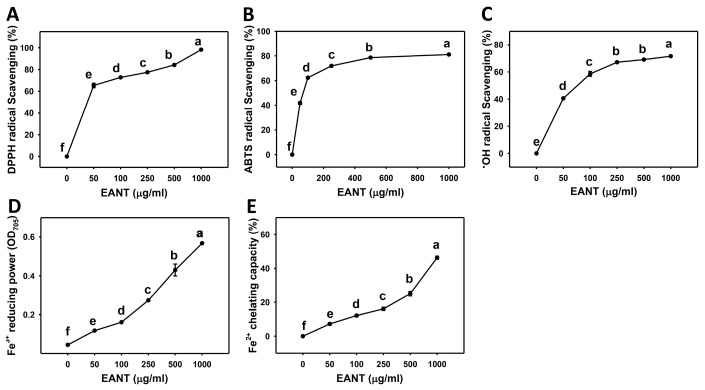
Concentration effect of EANT on the scavenging activities of DPPH, ABTS, and hydroxyl radical (^•^OH), as well as FRAP and FCP statuses. The concentration of EANT was shown as indicated in each panel (**A**–**E**). Data, mean ± SD (*n* = 3). For multiple comparisons, data without the same letters reveal a significant difference (*p* < 0.05–0.0001). For example (Figure 1C), the ^•^OH radical scavenging (%) at 250 and 500 μg/mL EANT show “b and b” indicating nonsignificant differences between each other because they overlap with the same lower-case letters. Similarly, the ^•^OH radical scavenging (%) at 50 and 250 μg/mL EANT showing “d” and “b” indicate significant differences among each other.

DPPH, ABTS, hydroxyl radical, FRAP, and FCP assays were used to determine the antioxidant potential of the EANT extract. Figure 1A shows the mean percentage DPPH scavenging activities of EANT treatment, which are 0, 65.37, 72.74, 77.44, 84.19, and 98.19%. Figure 1B shows the mean ABTS^•+^ radical scavenging activities of EANT treatment, which are 0, 41.86, 62.34, 71.87, 78.59, and 81.09%. Figure 1C shows the mean hydroxyl radical scavenging activities of EANT treatment, which are 0, 40.61, 58.57, 67.13, 69.06, and 71.55%. Accordingly, EANT dose-dependently increased all three, the DPPH, ABTS^•+^, and hydroxyl radical scavenging activities.

Figure 1D shows the mean FRAP (OD_705_) values of EANT treatment, which are 0.045, 0.118, 0.161, 0.273, 0.43, and 0.567. Figure 1E shows the mean FCP values of EANT treatment, which are 0, 7.19, 12.11, 16.07, 25.07, and 46.27%. Accordingly, EANT dose-dependently increased the FRAP and FCP activities.

### 3.2. EANT Decreases Cell Viability of Leukemia Cells

**Figure 2:**
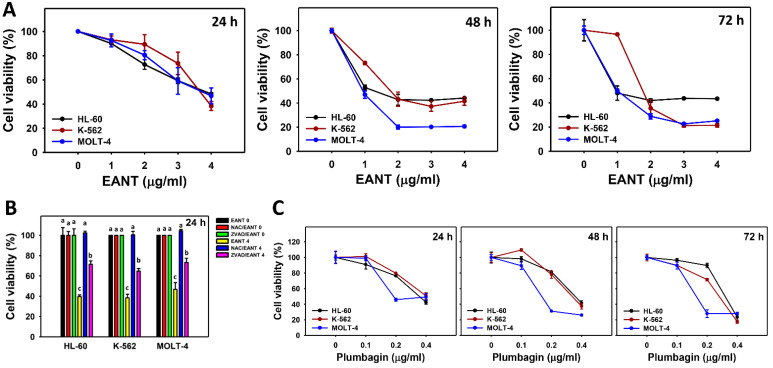
Leukemia cell viabilities following EANT treatment. (**A**) MTS assay at 24, 48, and 72 h. (**B**) Pretreatment impact of NAC and ZVAD on cell viability. Cells (HL-60, K-562, and MOLT-4) were pretreated with NAC (5 mM, 1 h), ZVAD (100 μM, 2 h), or instead post-treated with EANT (0 and 4 μg/mL for 24 h). The 0 μg/mL solution contained no EANT but contained 0.1% DMSO. (**C**) MTS assay at 24, 48, and 72 h for the main isolated compound plumbagin in EANT. Data, mean ± SD (*n* = 3). Data showing different alphabets at the top revealed a significant difference (*p* < 0.0001 for multiple comparisons). In the example of HL-60 cells (Figure 2B), the EANT 0, NAC/EANT 0, ZVAD/EANT 0, and NAC/EANT 0 show “a”, indicating nonsignificant differences between each other because they overlap with the same lower-case letters. Similarly, the NAC/EANT 0, ZVAD/EANT 4, and EANT 4 showing “a, b, and c” indicate significant differences among each other.

According to Figure 2, the cell viabilities (%) of leukemia cells (HL-60, K-562, and MOLT-4) were decreased upon EANT treatment at 24, 48, and 72 h. In addition, the function of oxidative stress and apoptosis in inhibiting the proliferation of EANT in leukemia cells was assessed by their inhibitors, such as NAC and ZVAD. At 24 h of treatment with 4 μg/mL EANT, all antiproliferation effects of EANT on the three leukemia cell lines were converted to normal proliferation by NAC and moderately reverted by ZVAD.

Moreover, the cytotoxic effect of the main isolated compound plumbagin in EANT [12] was determined on these three leukemic cell lines. The cell viabilities (%) of leukemia cells (HL-60, K-562, and MOLT-4) were decreased upon compound treatment at 24, 48, and 72 h (Figure 2C). These results suggested that plumbagin may participate in the antiproliferation effects of EANT in leukemia cells.

### 3.3. EANT Increases Populations for SubG1 and G1 Phases in Leukemia Cells

**Figure 3:**
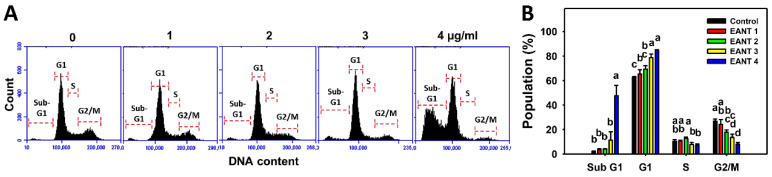
Cell cycle change effects of EANT-treated leukemia cells. (**A**,**B**) Histogram and statistical analysis for cell cycle distribution. Leukemia cells (HL-60) were treated with EANT (24 h, 0 to 4 μg/mL). The 0 μg/mL solution means no EANT but contains 0.1% DMSO. EANT 1, 2, 3, and 4 indicate EANT 1, 2, 3, and 4 μg/mL, respectively. Data, means ± SD (*n* = 3). Data showing different alphabets at the top reveal a significant difference (*p* < 0.05–0.0001 for multiple comparisons).

Figure 3A demonstrates the histograms for cell cycle distribution in EANT-treated leukemia HL-60 cells. In Figure 3B, EANT dose-dependently causes more subG1 and G1 populations than the control in leukemia HL-60 cells. Since 4 μg/mL EANT showed a dramatic induction of the subG1 population (an apoptosis-like status), this concentration was chosen to apply to the following time-course experiments to explore the mechanisms involving oxidative stress, apoptosis, and DNA damage.

### 3.4. EANT Caused Apoptosis in Leukemia Cells

**Figure 4:**
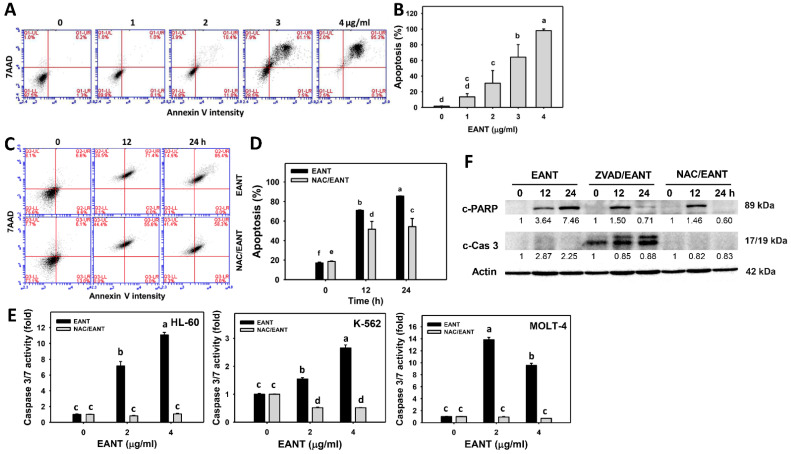
Annexin V status of EANT-treated leukemia cells. (**A**,**B**) Histogram and statistical analysis for annexin V expression. Apoptosis (%) was calculated to the populations of annexin V (+)/7AAD (+/−) (%). HL-60 cells were treated with EANT (24 h, 0 to 4 μg/mL). The control contained 0 μg/mL EANT but 0.1% DMSO. (**C**,**D**) Histogram and statistical analysis for annexin V detection of HL-60 cells following NAC/EANT treatment. NAC/EANT indicates that cells are either pretreated with NAC (5 mM, 1 h) or post-treated with EANT (0 and 4 μg/mL for 0, 12, and 24 h). (**E**) Caspase 3/7 activity assay of HL-60, K-562, and MOLT-4 cells following NAC/EANT treatment for 1 h/24 h. Data, mean ± SD (*n* = 3). Data showing different letters at the top revealed a significant difference (*p* < 0.05–0.0001 for multiple comparisons). (**F**) Western blot analysis of c-PARP and c-Cas 3 expressions in HL-60 cells following NAC (5 mM, 1 h)/EANT or ZVAD (100 μM, 2 h)/EANT treatments.

The subG1 increasing effect, as shown in Figure 3, indicates an apoptosis-like response. To further validate the apoptosis effect of EANT, an annexin V/7AAD analysis was performed. Figure 4A demonstrates the annexin V histograms for EANT-treated leukemia HL-60 cells. In Figure 4B, leukemia HL-60 cells following EANT treatment exhibit higher annexin V (+) cells than the control in a dose-dependent manner.

Since NAC recovered EANT-induced antiproliferation (Figure 2), we assessed the NAC effect on apoptosis in leukemia cells. Figure 4C shows the annexin V histograms for EANT-treated leukemia cells with and without NAC pretreatment. In Figure 4D, leukemia HL-60 cells cause more annexin V (+) populations than the control at various time intervals, which is suppressed by NAC.

Cas 3/7 activity assays were further applied to detect the expected caspase activity of apoptosis. In Figure 4E, the caspases 3/7 activities of HL-60, K-562, and MOLT-4 cells are upregulated by EANT at 24 h of treatment, and they are suppressed by NAC pretreatment. In Figure 4F, EANT shows higher c-PARP and c-Cas 3 expressions than the control in a Western blot analysis of leukemia cells. The apoptosis protein c-PARP and c-Cas 3 expressions were suppressed by NAC and ZVAD pretreatment, especially at 24 h of EANT treatment.

### 3.5. EANT Caused ROS Induction in Leukemia Cells

**Figure 5:**
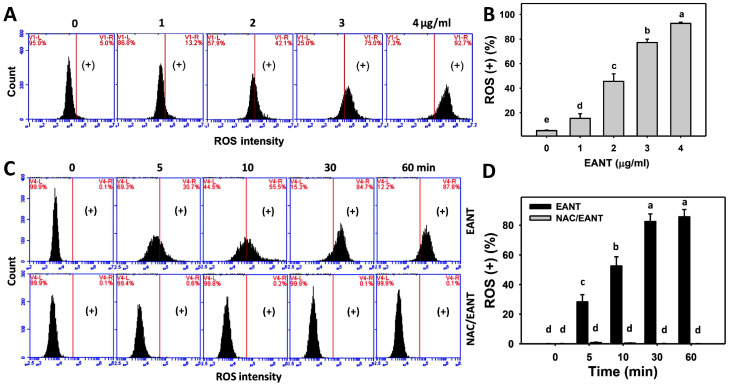
ROS content of EANT-treated leukemia cells. (**A**,**B**) Histogram and statistical analysis for ROS content. (+) at the right side of each panel is regarded as the population of ROS (+) (%). HL-60 cells were treated with EANT (1 h, 0 to 4 μg/mL). The control contained 0 μg/mL EANT but 0.1% DMSO. (**C**,**D**) Histogram and statistical analysis for ROS content of HL-60 cells following NAC/EANT treatment. NAC/EANT indicates that cells are either pretreated with NAC (5 mM, 1 h) or post-treated with EANT (0 and 4 μg/mL for 0 to 60 min). Data, mean ± SD (*n* = 3). Data showing different alphabets at the top reveal a significant difference (*p* < 0.05–0.0001 for multiple comparisons).

The contribution of oxidative stress in EANT-treated leukemia cells was investigated by ROS monitoring. Figure 5A shows the ROS histograms for EANT-treated leukemia HL-60 cells. Figure 5B shows that leukemia HL-60 cells following EANT treatment exhibit more ROS (+) populations than the control in a dose-dependent manner.

Since NAC recovered EANT-induced antiproliferation (Figure 1), the NAC effect on ROS induction in leukemia cells was assessed. Figure 5C demonstrates the ROS histograms for EANT-treated leukemia cells with and without NAC pretreatment. In Figure 5D, leukemia HL-60 cells cause more ROS (+) populations than the control at various time intervals, which is suppressed by NAC.

### 3.6. EANT Causes Superoxide Induction in Leukemia Cells

**Figure 6:**
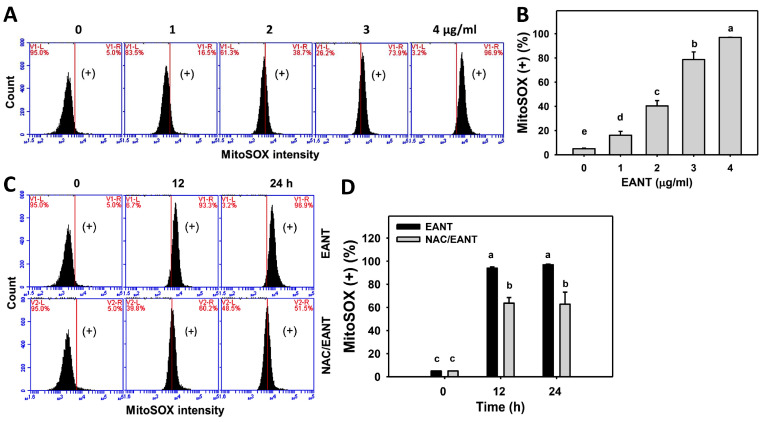
MitoSOX content of EANT-treated leukemia cells. (**A**,**B**) Histogram and statistical analysis for MitoSOX content. (+) in the right side of each panel is regarded as the population of MitoSOX (+) (%). HL-60 cells were treated with EANT (24 h, 0 to 4 μg/mL). The control contained 0 μg/mL EANT but 0.1% DMSO. (**C**,**D**) Histogram and statistical analysis for MitoSOX content of HL-60 cells following NAC/EANT treatment. NAC/EANT indicates that cells are either pretreated with NAC (5 mM, 1 h) or post-treated with EANT (0 and 4 μg/mL for 0, 12, and 24 h). Data, mean ± SD (*n* = 3). Data showing different letters on the top reveal a significant difference (*p* < 0.05–0.0001 for multiple comparisons).

The involvement of oxidative stress of leukemia following EANT was addressed by MitoSOX monitoring. Figure 6A demonstrates the MitoSOX histograms for EANT-treated leukemia HL-60 cells. In Figure 6B, leukemia HL-60 cells following EANT treatment exhibit more MitoSOX (+) populations than the control in a dose-dependent manner.

Since NAC recovered EANT-induced antiproliferation (Figure 1), the NAC effect on MitoSOX induction in leukemia cells was assessed. Figure 6C demonstrates the MitoSOX histograms for EANT-treated leukemia cells with or without NAC pretreatment. In Figure 6D, leukemia HL-60 cells cause more MitoSOX (+) populations than the control at various time intervals, which is suppressed by NAC.

### 3.7. EANT Causes MMP Dysfunction in Leukemia Cells

**Figure 7:**
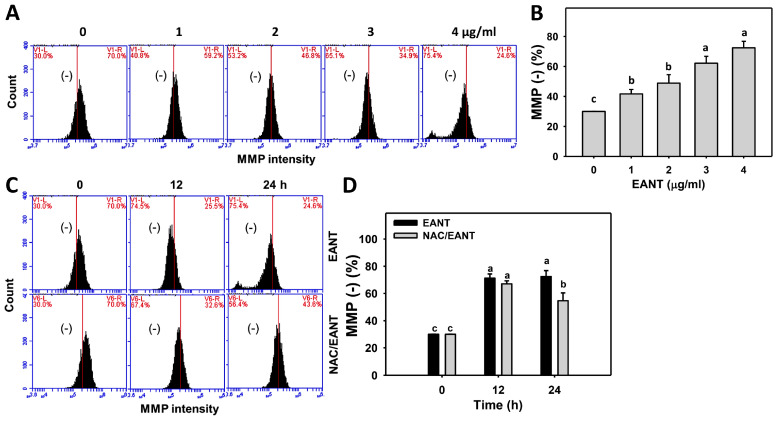
MMP content of EANT-treated leukemia cells. (**A**,**B**) Histogram and statistical analysis for MMP content. (−) on the left side of each panel is regarded as the population of MMP (−) (%). HL-60 cells were treated with EANT (1 h, 0 to 4 μg/mL). The 0 μg/mL solution meant no EANT but contained 0.1% DMSO. (**C**,**D**) Histogram and statistical analysis for MMP content of HL-60 cells following NAC/EANT treatment. NAC/EANT indicates that cells are either pretreated with NAC (5 mM, 1 h) or post-treated with EANT (0 and 4 μg/mL for 0, 12, and 24 h). Data, mean ± SD (*n* = 3). Data showing different letters on the top reveal a significant difference (*p* < 0.05–0.0001 for multiple comparisons).

The function of oxidative stress of leukemia following EANT was addressed by MMP monitoring. Figure 7A demonstrates the MMP histograms for EANT-treated leukemia HL-60 cells. In Figure 7B, leukemia HL-60 cells following EANT treatment exhibit more MMP (−) populations than the control in a dose-dependent manner.

Since NAC recovered EANT-induced antiproliferation (Figure 1), the NAC effect on MMP dysfunction in leukemia cells was assessed. Figure 7C demonstrates the MMP histograms for EANT-treated leukemia cells with and without NAC pretreatment. In Figure 7D, leukemia HL-60 cells cause more MMP (−) populations than the control at various time intervals, which is suppressed by NAC.

### 3.8. EANT Causes Antioxidant Gene Expressions in Leukemia Cells

**Figure 8:**
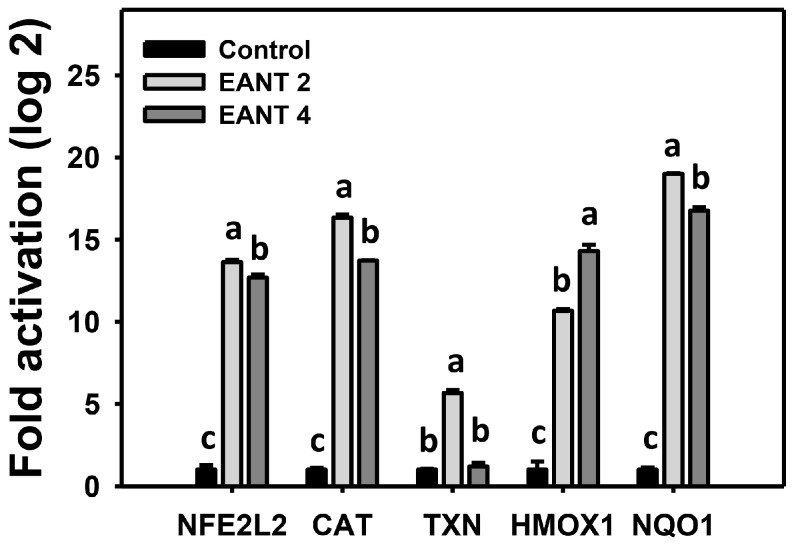
mRNA expressions for antioxidant response in EANT-treated leukemia cells. Several genes (*NFE2L2*, *CAT*, *TXN*, *HMOX1*, and *NQO1*) were chosen for antioxidant signaling. HL-60 cells were treated with 0, 2, and 4 μg/mL EANT (control, EANT 2, and EANT 4) for 24 h. The relative expression is represented as fold activation (log_2_). Data, mean ± SD (*n* = 3). Data showing different letters on the top reveal a significant difference (*p* < 0.05–0.0001 for multiple comparisons of the same gene).

The antioxidant system is modulated by oxidative stress [41]. mRNA expressions for antioxidant genes [38,42,43], including *NFE2L2*, *CAT*, *TXN*, *HMOX1*, and *NQO1,* were monitored by real-time RT-PCR analysis following EANT treatment in HL-60 cells. These antioxidant genes were upregulated by EANT (Figure 8).

### 3.9. EANT Causes γH2AX Type of DNA Damages in Leukemia Cells

**Figure 9:**
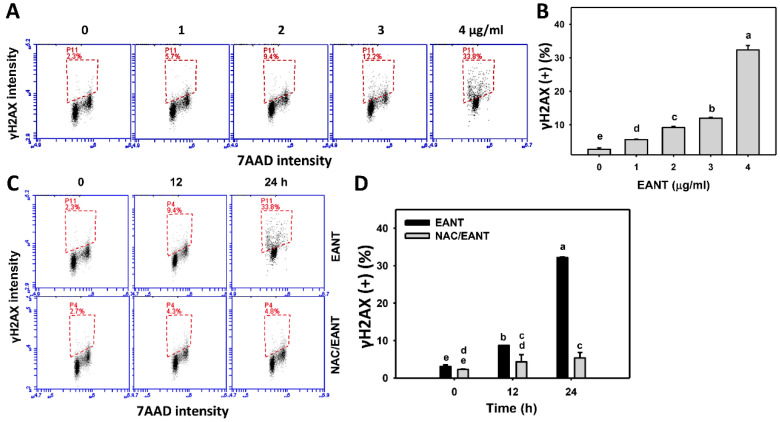
γH2AX content of EANT-treated leukemia cells. (**A**,**B**) Histogram and statistical analysis for γH2AX content. The dash-lined region of each panel is regarded as the population of γH2AX (+) (%). HL-60 cells were treated with EANT (1 h, 0 to 4 μg/mL). The control contained 0 μg/mL EANT but 0.1% DMSO. (**C**,**D**) Histogram and statistical analysis for γH2AX content of HL-60 cells following NAC/EANT treatment. NAC/EANT indicates that cells were either pretreated with NAC (5 mM, 1 h) or post-treated with EANT (0 and 4 μg/mL for 0, 12, and 24 h). Data, mean ± SD (*n* = 3). Data showing different alphabets at the top reveal a significant difference (*p* < 0.05–0.0001 for multiple comparisons).

γH2AX is a DNA damage marker for double-strand breaks. The change in the DNA damage of leukemia following EANT was addressed by γH2AX monitoring. Figure 9A shows the γH2AX histograms for EANT-treated leukemia HL-60 cells. In Figure 9B, leukemia HL-60 cells following EANT treatment exhibit more γH2AX (+) populations than the control in a dose-dependent manner.

Since NAC recovered EANT-induced antiproliferation (Figure 1), the NAC effect on the γH2AX type of DNA damage in leukemia cells was assessed. Figure 9C demonstrates the γH2AX histograms for EANT-treated leukemia cells with and without NAC pretreatment. In Figure 9D, leukemia HL-60 cells cause more γH2AX (+) populations than the control at various time intervals, which is suppressed by NAC.

### 3.10. EANT Causes 8-OHdG Type of DNA Damages in Leukemia Cells

**Figure 10:**
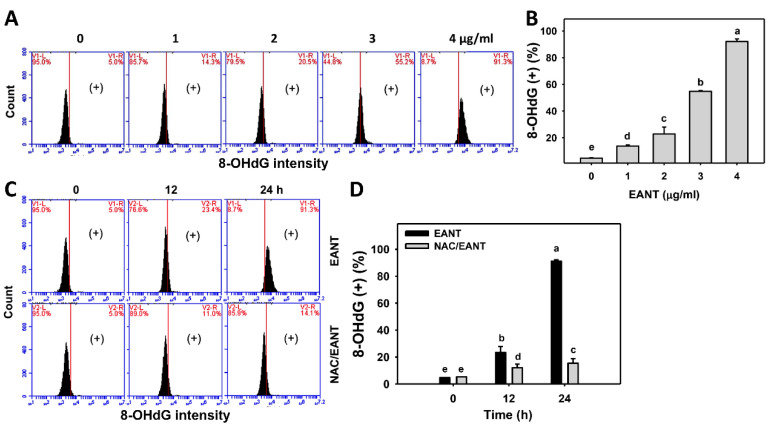
8-OHdG content of EANT-treated leukemia cells. (**A**,**B**) Histogram and statistical analysis for 8-OHdG content. (+) on the right side of each panel is regarded as the population of 8-OHdG (+) (%). HL-60 cells were treated with EANT (1 h, 0 to 4 μg/mL). The control contained 0 μg/mL EANT but 0.1% DMSO. (**C**,**D**) Histogram and statistical analysis for 8-OHdG content of HL-60 cells following NAC/EANT treatment. NAC/EANT indicates that cells were either pretreated with NAC (5 mM, 1 h) or post-treated with EANT (0 and 4 μg/mL for 0, 12, and 24 h). Data, mean ± SD (*n* = 3). Data showing different letters on the top reveal a significant difference (*p* < 0.0001 for multiple comparisons).

8-OHdG is a marker for oxidative DNA damage. The change in the oxidative DNA damage of leukemia following EANT was addressed by 8-OHdG monitoring. Figure 10A demonstrates the 8-OHdG histograms for leukemia cells for EANT-treated leukemia HL-60 cells. In Figure 10B, leukemia HL-60 cells following EANT treatment develop more 8-OHdG (+) populations than the control in a dose-dependent manner.

Since NAC recovered EANT-induced antiproliferation (Figure 1), the NAC effect on the 8-OHdG type of DNA damage in leukemia cells was assessed. Figure 10C demonstrates the 8-OHdG histograms for EANT-treated leukemia cells with and without NAC pretreatment. In Figure 10D, leukemia HL-60 cells cause more 8-OHdG (+) populations than the control at various time intervals, which is suppressed by NAC.

## 4. Discussion

The anticancer effect of EANT, ethyl acetate extract of *N. thorelii* x *(ventricosa* x *maxima)*, has only been reported in breast cancer cells [12], and its antioxidant ability and anticancer effect on leukemia cells remain unclear. In the present study, we assessed the antioxidant ability using five standard antioxidant assays, antiproliferation by MTS assay, and detailed mechanisms by flow cytometry and Western blotting analysis. Several connections between each finding for EANT-treated leukemia cells are discussed below.

As yet, anti-leukemia reports for Nepenthes extracts are rare according to a PubMed search. AML was developed from myeloid cells, while ALL was developed from different types of lymphocytes (B- or T-cells). Both AML and ALL types of leukemia cells may not be metabolically homogeneous and exhibit different metabolic characteristics [44]. For example, AML and T-ALL cell lines show higher glycolytic and cell respiration gene expressions than those of B-ALL cell lines [45]. It is, therefore, interesting to examine the drug responses to EANT on AML (HL-60 and K-562) and ALL (MOLT-4) cell lines. In the current study, IC_50_ values of EANT-treated AML HL-60 and K-562 and ALL MOLT-4 types of leukemia cells were 3.85, 3.68, and 3.73 µg/mL for the 24 h MTS assay, 1.28, 1.76, and 0.94 μg/mL for the 48 h MTS assay, and 0.96, 1.76, and 0.99 for the 72 h MTS assay, respectively. The time-dependent cytotoxicity of EANT shows at HL-60 cells from 24 to 72 h and at K-562 and MOLT-4 cells from 24 to 48 h. For breast cancer MCF7 and SKBR3 cells, their IC_50_ values of EANT were 15 and 25 µg/mL following a 24 h MTS assay [12]. Accordingly, leukemia cells showed about 3.5–6.5 fold higher sensitivity to EANT than breast cancer cells did.

The bioactive compounds of EANT, including plumbagin, *cis*-isoshinanolone, and quercetin 3-*O*-(6″-*n*-butyl β-D-glucuronide), were reported previously by high-performance liquid chromatography (HPLC) analysis [12]. Except for *cis*-isoshinanolone [46], quercetin 3-*O*-(6″-*n*-butyl β-D-glucuronide) exhibits anticancer effects on liver and breast cancer cells [47], and plumbagin shows anticancer effects on several types of cancer cells [48]. For the ALL type of leukemia cells (MOLT-4), plumbagin shows an IC_50_ value of 0.19 μg/mL for the 24 h CCK-8 assay, but it shows no cytotoxicity to normal peripheral blood mononuclear cells [49]. For the AML type of leukemia cells (Kasumi1 and HL-60), plumbagin shows IC_50_ values of 0.85 and 0.28 μg/mL for the 24 h CCK-8 assay [50] and the 48 h MTT assay [51]. Similarly, plumbagin shows IC_50_ values of 0.35, 0.4, and 0.19 μg/mL for 24 h; 0.35, 0.33, and 0.16 μg/mL for 48 h; and 0.32, 0.27, and 0.16 μg/mL for 72 h MTS assays of leukemia cells (HL-60, K-562, and MOLT-4, respectively) (Figure 2C). Further evaluation for the anticancer effects of the other EANT-derived compounds on leukemia cells is warranted. Moreover, the proliferation effect of normal cells on EANT needs further examination.

Plumbagin targets the transactivation domain of Myb to suppress Myb activity [52]. In addition, five potential targets of plumbagin were reported, namely phosphatidylinositol-4,5-bisphosphate 3-kinase (PI3Kγ), AKT1, Bcl-2, nuclear factor kappa B subunit (NF-κB), and signal transducer and activator of transcription 3 (STAT3), using molecular docking and (un)binding simulation analysis [53]. Making use of the differentiation-inducing effect is a promising strategy for the treatment of AML. Plumbagin also shows a differentiation-inducing effect on AML HL-60 cells [52]. Moreover, plumbagin can inhibit the proliferation of primary AML cells derived from patients but not for normal hematopoietic progenitors. Therefore, further evaluation of the differentiation-inducing effects of EANT and other EANT-derived compounds on leukemia cells is warranted in the future.

For cellular redox homeostasis, exogenous antioxidants may provide a bi-functional regulation of oxidative stress. They decrease oxidative stress at physiological concentrations but increase oxidative stress at high concentrations [20]. Similarly, ROS-inducing agents [31], natural products, and herbal medicines [54,55] show anticancer effects by generating exogenous ROS to exceed the tolerance of redox homeostasis in cancer cells [56].

*Nepenthes* species adapt to diverse eco-geographical conditions and exhibit distinct metabolite regulation patterns [57]. Interestingly, several *Nepenthes* extracts commonly exhibit antioxidant effects. For example, the methanol/chloroform/water extracts of *N. ampullaria*, *N. rafflesiana*, and *N. hookeriana* show antioxidant abilities for DPPH, FRAP, and total phenolic content. In addition, the methanol extracts of *N. khasiana* [58] and *N. bicalcarata* [9] show antioxidant abilities for DPPH, ABTS, and FRAP. Similarly, EANT provides antioxidant activities for DPPH, ABTS, hydroxyl radical, FRAP, and FCP.

The oxidative stress-inducing effect of EANT was validated by the evidence of ROS, MitoSOX, and MMP flow cytometry in leukemia HL-60 cells (Figure 5, Figure 6 and Figure 7). Moreover, mitochondrial metabolism is a vital target for AML therapy [59]. Targeting mitochondria for ROS and MitoSOX modulations can improve the therapeutic effects of AML [23,60]. Similarly, natural products, such as *Rosa cymose* fruits, exhibit DPPH antioxidant ability and have ROS-inducing potential in leukemia cells [23]. Some marine sponge-derived natural products also demonstrate both an antioxidant ability and antiproliferation of cancer cells [61,62].

Antioxidant gene expression and oxidative stress have a cross-talk interaction [63,64]. In response to sustained exogenous oxidative stress, NFE2L2 and its target TXN were activated [65]. CAT was also triggered by oxidative stress [66]. *CAT* and *HMOX1* mRNA and protein in mice were upregulated in response to UVC irradiation-induced oxidative stress [67]. Moreover, *NQO1* knockdown suppressed oxidative stress in prostate cancer cells [68]. Consistent with the present study, EANT enhanced the mRNA expressions of *NFE2L2*, *CAT*, *TXN*, *HMOX1,* and *NQO1* genes (Figure 8) in leukemia cells in response to EANT-induced oxidative stress. Therefore, the antioxidant capacity of EANT is probably associated with the antiproliferation response to leukemia cells through oxidative stress generation. It is noted that mRNA levels may not be in accordance with the protein levels of antioxidant signaling genes. A detailed investigation of antioxidant protein expressions of leukemia cells following EANT treatment is warranted in the future.

The toxic effect of high oxidative stress frequently induces apoptosis [69] and DNA damage [70] in cancer therapy. For example, the ethanol extract of *Rosa cymose* fruits upregulates ROS generation, disrupts MMP, induces a γH2AX type of DNA damage, and triggers apoptosis in leukemia cells [23]. As the responses of breast cancer cells [12], EANT also shows cellular (ROS) and mitochondrial (MitoSOX) oxidative stresses to trigger apoptosis and to induce a γH2AX type of DNA damage to leukemia cells. Moreover, oxidative DNA damage adduct 8-OHdG is also induced following EANT treatment.

Although apoptosis upon EANT treatment was reported in breast cancer cells [12], detailed apoptosis signaling was not investigated. In the present study, the apoptosis response was explored with the evidence of caspase 3/7 activations in leukemia HL-60 cells (Figure 4E). We also demonstrated that EANT induced c-PARP and c-Cas 3 expressions by a Western blot assay. However, other apoptosis-related signaling, such as p21, p53, B-cell lymphoma-2 (Bcl-2), and Bcl-2-associated X protein (Bax), were not investigated. c-Jun N-terminal protein kinase (JNK) can activate apoptosis signaling [71]. Extracellular signal-regulated kinase 1/2 (ERK 1/2) and AKT serine/threonine kinase (AKT) also regulates apoptosis. For example, adiponectin activates ERK 1/2 and AKT to inhibit neutrophil apoptosis [72]. Accordingly, a detailed examination of apoptotic pathway-related protein expressions, such as p21, p53, Bcl-2, Bax, JNK-1, ERK-1, and AKT, is warranted. Moreover, in our study, ZVAD recovers the EANT-induced antiproliferation (Figure 1B). Therefore, the role of apoptosis in antiproliferation was validated in the anti-leukemia effect of EANT.

EANT induces oxidative stress; however, the dependence of oxidative stress on all test changes in leukemia cells following EANT treatment needed to be examined. Using NAC pretreatment, the EANT-associated changes of cell viability, cell cycle dysregulation, cellular and mitochondrial oxidative stress, apoptosis, and DNA damage were reverted. Accordingly, the antiproliferation effect and mechanisms were mediated by oxidative stress in leukemia cells.

## 5. Conclusions

The antioxidant and antiproliferation properties have rarely been examined for *Nepenthes* ethyl acetate extract (EANT) in leukemia cells. In the present study, we firstly reported that EANT exhibits antioxidant abilities and demonstrates the antiproliferation of acute leukemia cells. With the pretreatment of ROS and apoptosis inhibitors, oxidative stress and apoptosis were validated to contribute to the antiproliferation of leukemia cells following EANT exposure. Mechanistically, EANT causes cell cycle disturbance associated with apoptosis expression and signaling and induces several oxidative stress changes and DNA damages in leukemia cells. ROS inhibitors alleviated all these EANT-induced changes. The weakness of the present study was the lack of some experiments on the three leukemia cell lines. Most results were demonstrated only using one leukemia cell type (HL-60) to investigate the possible antiproliferation mechanisms. In conclusion, EANT exhibits antiproliferation and apoptosis function on leukemia HL-60 cells relying on oxidative stress modulation.

## Data Availability

Data is contained within the article.
